# Genetic control of renal tumorigenesis by the mouse *Rtm1* locus

**DOI:** 10.1186/1471-2164-14-724

**Published:** 2013-10-22

**Authors:** José Ricardo Jensen, Antonella Galvan, Andrea Borrego, Wafa Hanna Koury Cabrera, Orlando Garcia Ribeiro, Nancy Starobinas, Marcelo De Franco, Maurizio Colecchia, Alessia Bertolotti, Tommaso Antonio Dragani, Olga Célia Martinez Ibañez

**Affiliations:** Laboratory of Immunogenetics, Instituto Butantan, Avenida Dr. Vital Brazil, 1500, 05503-900 São Paulo, Brazil; Department of Predictive and Preventive Medicine, Fondazione IRCCS Istituto Nazionale dei Tumori, Milan, Italy; Department of Pathology, Fondazione IRCCS Istituto Nazionale dei Tumori, Milan, Italy

**Keywords:** Animal models, Genetic linkage, Inflammation, Kidney cancer, QTL, SNPs

## Abstract

**Background:**

The genetic basis of susceptibility to renal tumorigenesis has not yet been established in mouse strains. Mouse lines derived by bidirectional phenotypic selection on the basis of their maximal (AIRmax) or minimal (AIRmin) acute inflammatory responsiveness differ widely in susceptibility to spontaneous and urethane-induced renal tumorigenesis. To map the functional loci modulating renal tumor susceptibility in these mice, we carried out a genome-wide genetic linkage study, using SNP arrays, in an (AIRmax x AIRmin)F2 intercross population treated with a single urethane dose at 1 week of age and phenotyped for renal tumors at 35 weeks of age.

**Results:**

AIRmax mice did not develop renal tumors spontaneously nor in response to urethane, whereas in AIRmin mice renal tumors formed spontaneously (in 52% of animals) and after urethane induction (89%). The tumors had a papillary morphology and were positive for alpha-methylacyl-CoA racemase and negative for CD10. By analysis of 879 informative SNPs in 662 mice, we mapped a single quantitative trait locus modulating the incidence of renal tumors in the (AIRmax x AIRmin)F2 intercross population. This locus, which we named *Renal tumor modifier QTL 1 (Rtm1*), mapped to chromosome 17 at 23.4 Mb (LOD score = 15.8), with SNPs rs3696835 and rs3719497 flanking the LOD score peak. The A allele of rs3719497 from AIRmin mice was associated with a 2.5-fold increased odds ratio for renal tumor development. The LOD score peak included the *Tuberous sclerosis 2* (*Tsc2*) gene which has already been implicated in kidney disease: loss of function by germline retroviral insertion is associated with spontaneous renal tumorigenesis in the Eker rat, and heterozygous-null *Tsc2*^*(+/-)*^ mice develop renal cystadenomas.

**Conclusions:**

We mapped *Rtm1* as a single major locus modulating renal tumorigenesis in a murine intercross population. Thus, the AIR mouse lines can be considered a new genetic model for studying the role of germline and somatic molecular alterations in kidney neoplastic disease.

## Background

In humans, kidney cancer is a complex disease comprising several different tumor types characterized by different somatic mutations and showing different histological features and clinical courses [1]. Risk factors include smoking, obesity and hypertension [2]. Genetic factors also underlie the predisposition to renal cancers, as familial aggregations have been reported and the risk of developing the disease for first-degree relatives of renal cancer patients is 1.7- to 2.6-fold higher than that of the general population [3]. Studies of families with hereditary kidney cancers have permitted the identification of mutations in several genes, including the TSC1 and TSC2 genes linked with tuberous sclerosis, a multi-system hamartoma syndrome (reviewed in [1]). In addition, genome-wide association analyses in large case–control studies have detected three loci, on chromosomes 2p21, 11q13.3, and 12p11.23 [4, 5], associated with an increased risk of renal cell carcinoma (RCC), the most common renal cancer in humans. Despite this progress, there is still much to unravel about the genetic susceptibility to this heterogeneous disease; the ability to study the genetics of renal cancer in a mouse model would therefore prove advantageous.

Until now, a mouse model suitable for studying the genetics of the inherited predisposition to renal tumor has not been described but, as we show here, does already exist. We previously developed the AIRmax and AIRmin mouse lines by phenotypically selecting, from a heterogeneous population, mice that had either an intense or a weak acute inflammatory response (AIR) [6]. The phenotypes used to select these strains were the number of infiltrated leukocytes and the protein content at the site of inflammation induced by the subcutaneous injection of a non-specific, non-immunogenic agent (polyacrylamide beads) [6]. After about 20 generations of selective breeding, AIRmax and AIRmin mouse lines presented extreme “maximal” and “minimum” phenotypes, respectively [7] and, at that limit, were considered to be homozygous for the quantitative trait loci (QTL) that control the acute inflammatory reaction. In addition to the inflammatory response, AIRmax and AIRmin mice differ in their susceptibility to several types of chemically induced tumors, in particular in the lung, skin and colon [8–10]. For example, adult AIRmax mice are resistant and AIRmin are susceptible to developing lung tumors in response to urethane [9], a multiorgan carcinogen with a short half-life (<1 h) [11]. Although the susceptibility of these mice to kidney tumors has not yet been studied, we were motivated to investigate their suitability as a model system for studying renal cancer genetics, especially because in humans the inflammatory response seems to play a role in the prognosis of renal cancer patients [12].

In the present study, a single dose of urethane injected in 7-day old mice induced, in addition to lung tumors, multiple renal tumors in AIRmin mice but not in AIRmax mice. The genetic basis for the different susceptibility of the two lines was investigated by genome-wide SNP genotyping followed by linkage analysis with renal tumor incidence in the pedigree of a large (AIRmax X AIRmin)F2 intercross population.

## Results

### Renal tumorigenesis in AIRmax and AIRmin mice and in interline crosses

**Figure 1:**
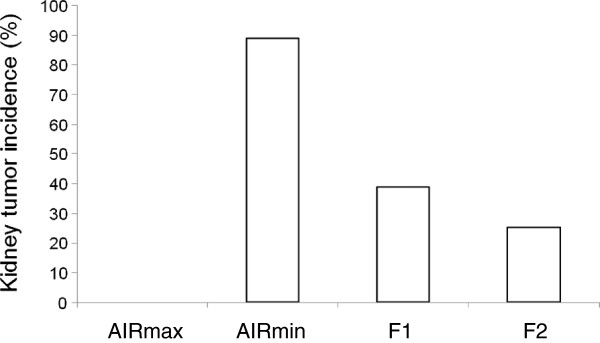
**Percent incidence of renal tumors in AIRmax (n = 15) and AIRmin (n = 9) mice and their F1 hybrid (n = 31) and F2 intercross (n = 662) offspring.** Mice were treated with 300 mg/kg urethane at 1 week of age and then followed until 35 weeks of age, when animals were sacrificed.

Untreated AIRmax mice (n = 15), when analyzed at 57 weeks of age, had no renal tumors, whereas 11 (52%) of 21 untreated AIRmin mice had spontaneous renal tumors at the same age. When mice pups were treated with urethane at 7 days and followed until 35 weeks of age, AIRmax mice remained resistant to renal tumorigenesis and did not develop any gross kidney tumor (Figure 1). In contrast, 8 (89%) of 9 urethane-treated AIRmin mice developed such tumors. Susceptibility to renal tumorigenesis was inherited as a co-dominant trait, since the incidence of renal tumors in urethane-treated (AIRmax x AIRmin)F1 mice was 39% while that in the (AIRmax x AIRmin)F2 intercross population was 25%. No statistically significant difference was observed in renal tumor incidence between male and female mice in any line.

**Figure 2:**
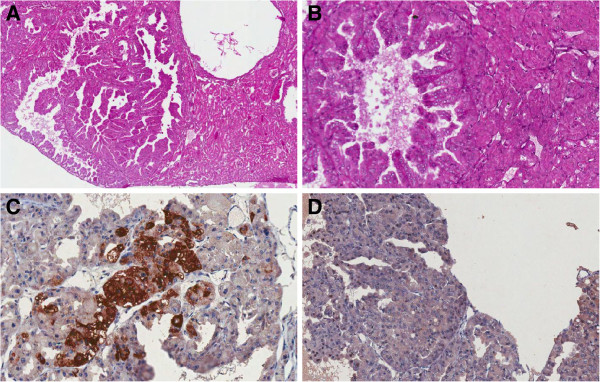
**Histological section of a papillary neoplastic proliferation of a kidney from an (AIRmax x AIRmin)F2 mouse.** Panel **A**, hematoxylin and eosin stained section, 40X magnification. Panel **B**, same section at 250X magnification. The renal tumor resulted positive for racemase (AMACR) immunostaining (panel **C**, 250X magnification) and negative for CD10 immunostaining (panel **D**, 250X magnification).

Tumor-bearing mice usually had multiple kidney tumors, which varied in size as seen both at gross examination and microscopically. Histopathological analysis of kidney tumors in selected F2 mice revealed the occurrence of lesions suggestive of papillary renal cell tumors, characterized by malignant epithelial cells constituting papillae and lining tubules (Figure 2A). Tumor-lined cysts with papillary excrescences were also seen (Figure 2B). Immunohistochemical analysis showed diffuse, marked staining for alpha-methylacyl-CoA racemase (AMACR) (Figure 2C), which in humans is a sensitive marker for papillary renal neoplasms. Staining for CD10, which is a marker of clear cell RCC and which is less frequently expressed by papillary tumors, was negative in our model (Figure 2D).

In addition, we carried out real-time PCR on RNA extracted from renal tumors and normal kidney tissue to measure the mRNA levels of known markers of these tumors. This analysis showed that the vimentin and β2-microglobulin genes were upregulated in renal tumors of F2 mice (n = 12) compared to normal renal tissue (not shown).

### One major locus controls renal tumor susceptibility in AIR mice

Of the 693 (AIRmax x AIRmin)F2 intercross mice available, genomic DNA and renal tumor phenotype were available for 662 animals. These mice were genotyped using SNP arrays representing a panel of 1449 SNPs. After quality control filtering of the genotype data, 879 markers (824 autosomal) resulted informative and were used in genome-wide linkage analysis. A statistical threshold for linkage with renal tumor incidence was determined by permutation analysis, resulting in a logarithm of odds (LOD) score of 3.7, for α=0.05 genome-wide statistical probabilities (10,000 permutations).

**Figure 3:**
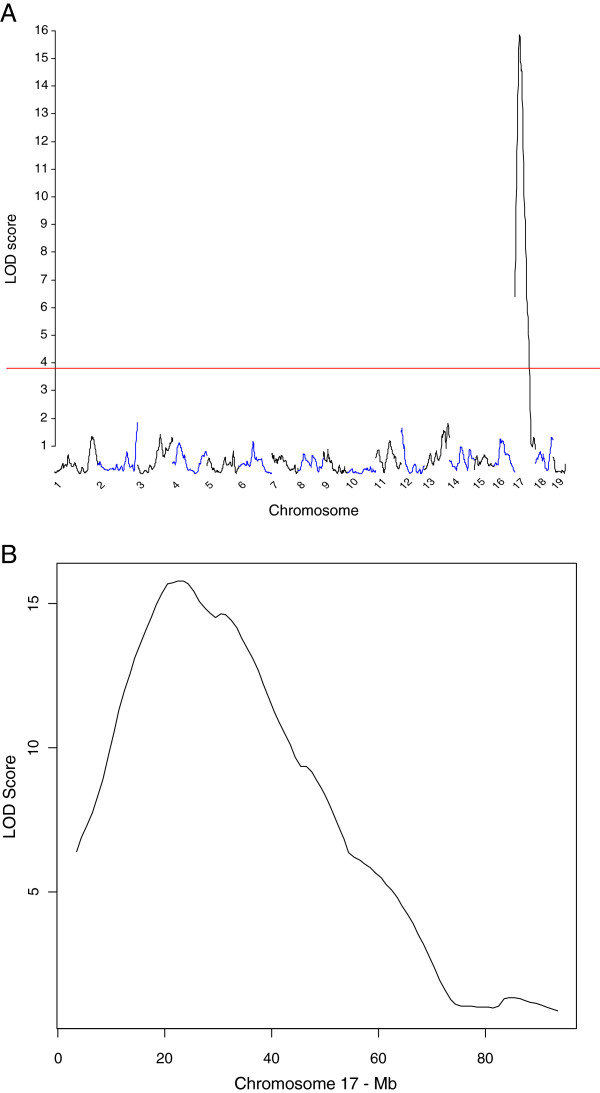
**Genome-wide genetic linkage analysis of renal tumor susceptibility in 662 (AIRmax x AIRmin)F2 intercross mice.** Panel **A**, above the genome-wide threshold value of LOD score = 3.67 for α=0.05 (horizontal red line), a single locus on chromosome 17 was detected. Panel **B**, details of chromosome 17 showing the mapping of the *Rtm1* locus, with LOD score peak of 15.8, at 23.4 Mb distance from the centromere.

Genome-wide linkage analysis detected a single QTL on chromosome 17, with a peak LOD score of 15.8, at 23.4 Mb (Figure 3A). The 1-LOD support interval was 11 Mb long, ranging from 18 to 29 Mb (Figure 3B). The SNPs flanking the LOD score peak were rs3696835 (22.681 Mb) and rs3719497 (25.502 Mb). We designated this locus *Renal tumor modifier QTL 1 (Rtm1*) associated with the incidence of renal tumors induced by urethane treatment. No other region of the genome showed a significant or suggestive association with the phenotype. Therefore, the *Rtm1* QTL likely harbors one or more genes with major effects in renal tumorigenesis.

**Figure 4:**
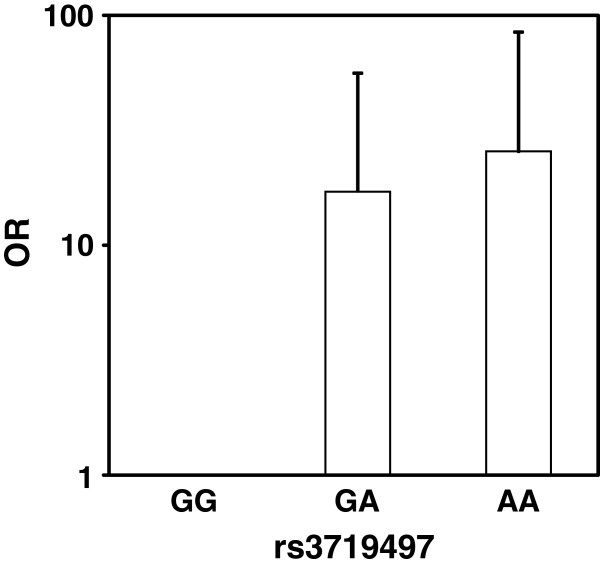
**Odds ratios (ORs) of renal tumor incidence of (AIRmax x AIRmin)F2 mice by genotype at rs3719497 on chromosome 17, located near the peak LOD score of genetic linkage between renal tumor incidence and genomic variants.** At the reference genotype GG, carried by AIRmax mice, renal tumors were observed in 4 of 163 F2 mice (2.5% incidence, reference category). At the GA genotype, renal tumors were seen in 110 of 361 mice (30.5%; OR = 14.4; 95% CI, 6.0 – 56.6), while at the AA genotype renal tumors were observed in 54 of 138 mice (39.1%; OR = 25.5; 95% CI, 8.5 – 86.1). ORs and 95% CIs are reported on a logarithmic scale.

Association analysis showed that, at SNP rs3719497, the G allele inherited from AIRmax mice confers resistance whereas the A allele from parental AIRmin was associated with a 2.5-fold increase in odds ratio (OR) for renal tumor development (P = 1.1 x 10^-12^, per allele OR = 2.5; 95% CI, 1.9 to 3.2). The ORs associated at each genotype in F2 mice showed a very strong association of homozygosity or heterozygosity of the AIRmin-derived A allele with renal tumor incidence (Figure 4). Homozygous inheritance of the AIRmin-derived allele at this locus caused a ~16–fold increase in the incidence of renal tumors (from 2.5% in heterozygotes to 30.5%). Similar findings were observed at the rs3696835, with the AIRmin-derived allele associated with an increased risk of renal tumors (not shown).

In the 1-LOD interval of the *Rtm1* locus, between 18 and 29 Mb, 378 known or predicted genes map^a^. Among these genes, the *Tsc2* gene, located within the LOD score peak, at 24.596-24.633 Mb, seems particularly relevant, due to its known involvement in renal tumorigenesis in rats, humans and genetically modified mice.

Genotypes of the 662 F2 mice were also assessed for association with the inflammatory response measured by the number of infiltrating cells (Ncell) at the local exudates induced by a subcutaneous injection of Bio-Gel P-100. The LOD score thresholds for linkage of Ncell, determined by permutation analysis, were 3.34 and 3.64, respectively, for α=0.1 and α=0.05 genome-wide statistical probabilities (5000 permutations). On chromosome 17, which contains the *Rtm1* locus, no loci had LOD scores above the genome-wide thresholds (not shown). This result indicates that *Rtm1* is independent of any loci associated with the inflammatory response.

## Discussion

Our findings show that AIRmin mice carry a co-dominant genetic susceptibility trait to renal tumorigenesis induced by urethane. By genome-wide linkage mapping of an (AIRmax x AIRmin)F2 intercross population, we found that such trait is regulated by germline genetic variations in a locus in the proximal region of chromosome 17. Within this locus, which we named *Rtm1*, the A allele of rs3719497 was associated with a 2.5-fold increase in OR for renal tumor development; homozygous inheritance of this allele from AIRmin mice increased the incidence of renal tumors by ~16–fold. The *Rtm1* locus is novel and unique, as no other loci modulating renal tumorigenesis have previously been mapped in mouse crosses.

No significant linkage was found between the inflammatory response and SNPs on chromosome 17. This finding indicates that the *Rtm1* locus was fixed during phenotypic selection of AIR mice, independently from loci modulating inflammatory response phenotypes. Such a phenomenon may be due to random genetic drift, as already proposed for skin tumorigenesis in selected mouse lines [13].

AIRmax and AIRmin mice are known to differ in susceptibility to chemically induced tumors [8–10], and here we show that they also differ in susceptibility to spontaneous tumors: in the absence of any carcinogenic treatment, >50% of AIRmin mice developed renal tumors at 57 weeks of age, whereas no AIRmax mouse did. Although we did not carry out a time-course analysis of spontaneous renal tumor incidence, we noticed that after urethane treatment of 1-week-old AIRmin mice, renal tumors were observed earlier (34 weeks after treatment) and with an increased incidence (~90%) as compared to spontaneous renal tumorigenesis in the same mouse line. In contrast, urethane treatment did not induce renal tumors in the genetically resistant AIRmax mice. Therefore, it seems that a correlation exists between strain susceptibility to spontaneous and urethane-induced renal tumorigenesis, as has already been observed for lung tumorigenesis in a meta-analysis of numerous inbred mouse strains [14]. Our results suggest that both spontaneous and urethane-induced renal tumors are controlled by the same genetic locus, i.e., *Rtm1*, although we cannot exclude that additional loci may control spontaneous renal tumor susceptibility in AIR mice.

Characterization of the renal tumors by immunohistochemistry showed the expression of AMACR, a peroxisomal and mitochondrial enzyme involved in the β-oxidation of branched-chain fatty acids and fatty acid derivatives. The AMACR gene has one of the highest expression levels in papillary RCC in humans [15]. The presence of CD10 (common acute lymphoblastic leukemia antigen or neprilysin), on the other hand, was not detected. This protein is a cell membrane neutral endopeptidase which participates in the post-secretory inactivation of inflammatory and vasoactive peptides [16]. In the diagnosis of renal tumors, CD10 is more often positive in clear cell RCC than in papillary RCC [17]. Real-time PCR pointed to the overexpression of vimentin and β2-microglobulin. Vimentin protein positivity has been reported in both clear cell and papillary RCCs in human samples [18], and β2-microglobulin overexpression has been detected in human RCC [19].

Spontaneous renal tumors are rare in inbred mouse strains [20, 21]. One exception is the BALB/c substrain BALB/cf/Cd, which has a 24%-70% incidence of kidney adenocarcinomas [22, 23], probably due to the insertion of the mouse mammary tumor virus into a not yet identified chromosomal domain that is implicated in kidney tumorigenesis [23]. Gene disruption is also known to cause spontaneous renal carcinoma in the Eker rat, where an endogenous retroviral element (the rat intracisternal A-particle) has integrated into an intron of the tumor suppressor gene tuberous sclerosis 2 (*Tsc2*) [24–26]. This intronic transposition leads to the production of aberrant *Tsc2* transcripts, causing a functional inactivation of the *Tsc2* gene which leads to renal tumorigenesis.

The murine *Tsc2* gene on chromosome 17 maps to the *Rtm1* locus peak identified in this study. This gene spans over 36.7 kb, contains 40 exons, generates a full length transcript of 6018 bp and encodes a protein of 1742 amino acids^b^. The protein, called tuberin or TSC2 (in humans), controls the mTOR (mammalian target of rapamycin) signaling pathway [27]. Of the 1150 known polymorphisms in this gene among inbred strains^c^; four are missense variants in exons whereas the others are found in introns or in the upstream or downstream regions where they might play a role in modulating transcript levels. The gene’s genomic location and involvement in renal tumorigenesis in the rat make *Tsc2* a good candidate for the AIR renal tumor locus. Interestingly, *Tsc2*^(+/-)^ mice develop renal cystadenomas [28]. Sequencing the *Tsc2* gene in AIRmax and AIRmin mice, as well as in mice of their F2 intercross, may reveal polymorphisms that, by influencing *Tsc2* expression or tuberin function, modulate the susceptibility of these mice to renal tumorigenesis.

Another candidate gene that maps close to *Tsc2* is the *Pkd1* gene, which encodes the transmembrane protein polycystin-1. Mutations leading to a loss of function at this gene cause autosomal dominant polycystic kidney disease in both humans [29] and mouse models [30]. Polycystin-1 is involved in cell adhesion and has been shown to inhibit cancer cell migration and invasion, leading to the suggestion that *Pdk1* is a tumor suppressor gene [31]. There is also a functional link between *Tsc2* and *Pkd1*: in human cells, TSC2 protein alternates from an inactive, phosphorylated form in the cytosol to an active, non-phosphorylated form at the plasma membrane bound to the intracellular, carboxy-terminal tail of polycystin-1; in this subcellular position, TSC2 protein activates the GTPase of Rheb, resulting in the inactivation of the mTOR pathway [27].

## Conclusions

Herein, we mapped *Rtm1* as a novel mouse modifier locus of chemically induced renal tumorigenesis in pedigrees of non-inbred mice. This locus spans the *Tsc2-Pkd1* region of chromosome 17, whose candidate genes are involved in renal cancer in animal models and humans. These results point to the possible involvement of polymorphisms in this region in the functional regulation of mouse renal tumorigenesis. Further analysis of the complete nucleotide sequence of the *Tsc2-Pkd1* region distinguishing the two AIR lines at the *Rtm1* QTL region described in this study will allow the identification of the functional variant responsible for the high susceptibility to spontaneous and urethane-induced renal tumorigenesis of AIRmin mice and may provide insight into new modulating mechanisms of renal tumorigenesis in other species, including humans.

## Methods

### Mice crosses

AIRmax and AIRmin lines (formally designated IBut:AIRH and IBut:AIRL, respectively, at the Institute for Laboratory Animals Research, Washington, DC, USA) and their crosses were developed and maintained at the animal facilities of the Laboratory of Immunogenetics of the Butantan Institute. For genome-wide linkage analysis, pedigrees were obtained by intercrossing AIRmax with AIRmin mice (both from the 48^th^ generation of the selective bi-directional breeding), which resulted in the generation of 66 F1 (AIRmax x AIRmin) mice. These F1 mice were intercrossed to generate 693 (AIRmax x AIRmin)F2 mice. Animal procedures were approved by the Institutional Animal Care and Use Committee of Butantan Institute and all animals received humane care according to the criteria outlined in the “Guide for the Care and Use of Laboratory Animals” [32].

### Renal tumor induction and inflammatory response assay

To chemically induce tumors, mice pups received a subcutaneous injection of urethane (300 mg/kg body weight) 7 days after birth. After 2 months, the same mice were tested for their acute inflammatory response by subcutaneous injection of 750 μl of a sterile suspension of 67% Bio-Gel P-100 (Bio-Rad, Hercules, CA, USA) (53 mg dry weight/ml) in phosphate-buffered saline. Local exudates were harvested 24 h later: the total cell count (Ncell) was determined using Malassez hemocytometer chambers [6].

Mice were sacrificed 240 days after urethane treatment (i.e. at 35 weeks of age) in a CO_2_ chamber, and tumors in internal organs were scored macroscopically. Samples of normal and tumor-containing kidneys were collected for immunohistochemical analyses and RNA extraction.

As a negative control for the chemical induction of tumors, untreated parental AIRmax and AIRmin mice were sacrificed at 400 days (i.e. 57 weeks of age) and examined for the presence of spontaneous tumors.

### Immunohistochemistry

Tumor-containing kidneys were fixed with formalin and embedded in paraffin. Sections (3 μm) were rehydrated and antigens were retrieved with Tris-EDTA buffer (pH = 9) using a PT Link instrument (Dako, Glostrup, Denmark). Immunohistochemical analysis was done using the EnVision FLEX kit (Dako). Briefly, after endogenous peroxidase blocking, slides were incubated in a humidified chamber for 30 min with polyclonal antisera to either alpha-methylacyl coenzyme A racemase or CD10 (Proteintech Group, Chicago, IL, USA) diluted 1:50. The signal was amplified using specific EnVision FLEX Rabbit Linker and colorimetric detection was completed with 3-3′-diaminobenzidine. Slides were counterstained with hematoxylin and mounted with Eukitt mounting medium (Bio-Optica, Milan, Italy).

### Analysis of gene expression by qRT-PCR

Total RNA was extracted from RNAlater™ preserved kidney tumors and RNA integrity was determined in the Agilent 2100 bioanalyzer (Agilent, Waldbronn, Germany). Primers were designed with Primer-BLAST (http://www.ncbi.nlm.nih.gov/tools/primer-blast/), with the exception of *B2m,* which were obtained from PrimerBank (ID 144227219b3, http://pga.mgh.harvard.edu/primerbank/). The 5′-3′ primer sequences were: *B2m* (F CCCCACTGAGACTGATACATACG and R CGATCCCAGTAGACGGTCTTG); *Vim* (F TGGTACAAGTCCAAGTTTGC and R CTCCGGTACTCGTTTGACT) and Ribosomal protein S29 (*Rps29,* F TCTACTGGAGTCACCCACGGAAGT and R TCAGTCGAATCCATTCAAGGTCGC). Reactions were run in a StepOnePlus™ real-time detector using the Fast SYBR® Green Master Mix (Applied Biosystems, Foster City, CA). Relative gene expression was calculated by the ΔΔCt method [33] using pooled normal kidney mRNA from AIRmax mice as calibrator and *Rps29* as reference gene.

### Genome-wide SNP genotyping

Genomic DNA was extracted from tail tips using the E.Z.N.A. Tissue DNA Kit (Omega Bio-Tek, Norcross, GA, USA) and quantified by fluorimetry (Invitrogen, Carlsbad, CA, USA). SNPs were genotyped in each mouse of the pedigree with the BeadArray technology (Illumina, San Diego, CA, USA), using the Mouse MD Linkage Panel, which permits analysis of 1449 SNP loci; bead chips were read in an iScan system (Illumina), as described [34]. Genome maps are based on Genome assembly GRCm38, Ensembl release 71.

### Statistical analysis

The search for QTLs was carried out with genome-wide linkage analysis between genotypes and phenotypes by interval mapping using GridQTL version 1.3.2. [35]. The significance thresholds of LOD scores for phenotype-genotype linkages were estimated by genome-wide permutation analysis. Odds ratios were calculated using logistic analysis. P-values were two-sided.

### Endnote

^a^http://www.ensembl.org/biomart - Accessed May 9, 2013.

^b^http://www.ensembl.org/Mus_musculus/Transcript/Summary?db=core;g=ENSMUSG00000002496;r=17:24595937-24632629;t=ENSMUST00000097373 - Accessed on April 18, 2013.

^c^http://www.ensembl.org/Mus_musculus/Gene/Variation_Gene/Table?db=core;g=ENSMUSG00000002496;r=17:24595937-24632629;t=ENSMUST00000097373 - Accessed on April 18, 2013.

## Abbreviations

AIR: Acute inflammatory response
AMACR: Alpha-methylacyl coenzyme A racemase
LOD: Logarithm of odds
QTL: Quantitative trait locus
Rtm1: Renal tumor modifier QTL 1
SNP: Single nucleotide polymorphism.
